# Synergistic effect of co-culture rhizosphere *Streptomyces*: A promising strategy to enhance antimicrobial activity and plant growth-promoting function

**DOI:** 10.3389/fmicb.2022.976484

**Published:** 2022-08-11

**Authors:** Jing Li, Lin Zhang, Gan Yao, Lixiang Zhu, Jingling Lin, Chengqiang Wang, Binghai Du, Yanqin Ding, Xiangui Mei

**Affiliations:** ^1^State Key Laboratory of Crop Biology, College of Agronomy, Shandong Agricultural University, Tai’an, China; ^2^College of life sciences, Shandong Agricultural University, Tai’an, China

**Keywords:** *Streptomyces*, co-culture, growth promotion, disease prevention, metabonomics

## Abstract

Rhizosphere *Streptomyces* is one of the important types of rhizosphere microorganisms that plays an important role in promoting plant growth and controlling plant diseases to maintain agricultural ecosystem balance and green ecological agriculture development as beneficial bacteria. Microbial co-culture simulates the complex biocommunity in nature, which has more advantages than the monoculture with a synergistic effect. As the key signal mediums of microorganisms, plants, and their interactions, microbial metabolites are of great significance in revealing their functional mechanism. In this study, two potential plant growth-promoting rhizobacteria, *Streptomyces albireticuli* MDJK11, and *Streptomyces alboflavus* MDJK44, were selected to explore the effects of co-culture and monoculture on plant growth promotion and disease prevention, and the metabolic material basis was analyzed by metabonomics. Results showed that *Streptomyces* MDJK11, MDJK44 monoculture, and co-culture condition all showed good growth promoting and antimicrobial effects. Moreover, as compared to the monoculture, the co-culture showed the advantage of a synergistic enhancement effect. LC-MS-based metabonomics analysis showed the metabolic material bases of *Streptomyces* for plant growth promotion and disease prevention were mainly plant hormone and antibiotics and the co-culture condition could significantly stimulate the production of plant hormone promoters and macrolide, cyclic peptide, and aminoglycoside antibiotics. The study proved that the co-cultures of *S. albireticuli* MDJK11 and *S. alboflavus* MDJK44 have great potential in crop growth promotion and disease prevention.

## Introduction

Rhizosphere microorganisms are known as new “organs” of plants because of their close interaction and symbiosis with plants, which play an important role in the advantages of environmental friendliness and sustainable utilization, and more and more attention has been paid to their research and application in agricultural production (Zhang et al., 2017; Bakker et al., 2018; Wei et al., 2019; Fasusi and Babalola, 2021). As one of the most important types, a large number of rhizosphere *Streptomyces* are beneficial microorganisms that are abundant in soil and plant rhizosphere. Rhizosphere *Streptomyces* play an important role in plant growth that can promote plant growth development, and induce plant disease resistance and stress tolerance. In terms of plant disease control, Rhizosphere *Streptomyces* can inhibit pathogens’ growth through various mechanisms, such as producing antibiotics, antimicrobial peptides, and induction of systemic resistance of plants (Traxler and Kolter, 2015; Newitt et al., 2019).

Microbial metabolites are the key media of interaction between the environment and biology, which contain a large number of natural products with novel structures and good function. It is of great significance to explore these metabolites for understanding their role and utilization (Traxler and Kolter, 2015). Among them, streptomyces metabolites play an important role in many fields, including drug development, environmental ecology, and chemical biology (Yadav et al., 2017). So far, 75% of antibiotics found are produced by *Streptomyces* and more than 120 substances are used in actual production (Doroghazi et al., 2014; Traxler and Kolter, 2015; Hutchings et al., 2019; Yu et al., 2020). As for plant fungal disease control, rhizosphere *Streptomyces* can not only produce antimicrobial metabolites with novel structure and stable properties, but also have a series of advantages, such as broad-spectrum, high-effective activity without cross-resistance. It can be seen that rhizosphere *Streptomyces* play important roles in plant growth and agricultural ecological development.

Metabonomics is an emerging omics technology in recent years, which could speculate the physiological and pathological mechanisms behind it by means of analyzing the global metabolite changes in different samples (Fu et al., 2022). Non-targeted metabonomics is mainly used to distinguish metabolite phenotypes and compare different metabolites. By a qualitative and quantitative analysis of metabolites in the biological system, it can reflect the total metabolite information to the greatest extent, then analyze their similarity, difference, and change trends, and make reasonable explanations (Chanana et al., 2017; Iqrar et al., 2021; Oyedeji et al., 2021; Ye et al., 2022). Barkal developed a method of microbial secondary metabonomics combined with liquid–liquid extraction separation to explore the microbial culture conditions, metabolites separation procedure, and novel metabolites discovery (Barkal et al., 2016). Therefore, metabonomics is a powerful tool to explore the material basis of microbial co-culture, plant growth promotion, and disease prevention.

Microorganisms live in complex microbial communities in the natural environment. Microorganisms, plants, and the environment interact, share and exchange metabolic processes and signals, and play the role of synergy or competitive antagonism (Dong et al., 2022; Fang et al., 2022). Obviously, the monoculture of a single strain has some limitations under typical laboratory fermentation conditions. In previous studies, a single strain was mostly cultured for biological control or plant growth promoters and metabolites research. Although it could play a certain role, it often needs to be combined with relevant chemicals and fertilizers to achieve the desired effect. Besides, *Streptomyces* are rich in biosynthetic gene clusters, most of which have not been explored or expressed (Nett et al., 2009; Vurukonda et al., 2020; Yu et al., 2020). Microbial co-culture simulates the complex biota state in nature that can activate silent gene clusters in strains through interaction to stimulate the production of defensive, protective, and nutritional metabolites with various structures (Marmann et al., 2014). A series of studies have shown that co-culture can induce the expression of silenced genes, produce new bioactive components, increase the yield and richness of metabolites, and improve the overall activity of fermentation products to a certain extent (Pettit, 2009; Priyanka et al., 2022). Maglangit’s research showed the co-culture of rhizosphere Streptomyces sp. MA37 and *Pseudomonas* sp. could induce the expression of the seemingly silent biosynthetic gene clusters and lead to the upregulation of several metabolites that were not previously observed in the monocultures of each strain (Maglangit et al., 2020). By the mixed culture of *Streptomyces tendae* KMC006 and *Gordonia* sp. KMC005, a new acyclic polyene polyketide substituted with a β-D-digitoxopyranose was isolated with antimicrobial activity against *Micrococcus luteus* and *Enterococcus hirae* (Park et al., 2017). It can be seen that microbial co-culture is a simple and effective way to discover novel secondary metabolites and improve metabolites yield with many advantages. To better promote plant growth and improve plant stress resistance, and biological control effect, co-culture has become a new trend with great research potential and space.

**FIGURE 1:**
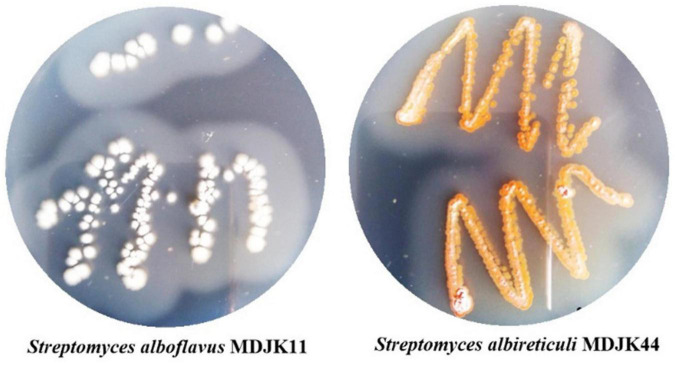
Morphological characteristics of *Streptomyces alboflavus* MDJK11 and *Streptomyces albireticuli* MDJK44.

According to a previous report, two *Streptomyces* species, *Streptomyces albireticuli* MDJK11, and *Streptomyces albofavus* MDJK44 (Figure 1), were isolated from the rhizosphere soil of peony. The complete genomic sequence analysis showed that the two strains were potential plant growth-promoting rhizobacteria with rich and novel secondary metabolic biosynthesis gene clusters (Wang et al., 2018). Herein, as our ongoing research, the above two rhizosphere *Streptomyces* have been proved to have certain plant growth promoting and antimicrobial activities. Interestingly, the co-culture of the two strains could enhance their growth promoting and antimicrobial effects that could play a synergistic role. We further explored the material basis of promoting the synergy of biocontrol and co-culture based on microbial metabonomics. In conclusion, the research shows that the two rhizosphere *Streptomyces* have the effect of growth promotion, microbial inhibition, and disease prevention, and co-culture can enhance the above functions with synergistic effects, which take plant hormones and antibiotics as their partial metabolic material basis. The research provides a scientific basis for further exploration of the two *Streptomyces* and provides references for the co-culture of microorganisms.

## Materials and methods

### Experimental materials

*Streptomyces albireticuli* MDJK11 and *S. albofavus* MDJK44 were isolated from the rhizosphere soil of peony (Wang et al., 2018). The standard indicator strain used in the experiment included *Escherichia coli* (ATCC 35218), *Bacillus subtilis* 168, *Fusarium oxysporum*, *Fusarium moniliforme*, *Fusarium solani*, and *Fusarium graminearum*, which were preserved in the research group. Plant species for disease promotion and prevention are Shan nong 27 wheat, Denghai 605 maize, and yellow flower tobacco seeds sold in Tai’an.

### Fermentation and crude extracts preparation of *Streptomyces*

*Streptomyces* MDJK11 and MDJK44 were well-cultured with GI medium (20.0 g soluble starch, 1.0 g KNO_3_, 0.5 g K_2_HPO_4_, 0.5 g MgSO_4_, 0.001 g FeSO_4_, and 0.5 g NaCl dissolved in 1.0 L distilled water) in 28°C for 3 days and then inoculated into the different culture medium (167 mL medium/500 mL flask). The flasks were continuously cultivated in a shaker with 300 revolutions per minute (rpm) and 30°C for 3 days as seed liquid. One milliliter of seed liquid was inoculated into a 167 mL medium/500 mL flask and then inoculated for 8 days when large-scale fermentation was performed. As for *Streptomyces* co-culture, 1 mL each of MDJK11 and MDJK44 seed liquid was inoculated into 167 mL medium/500 mL flask and then cultivated under the same conditions. Each experiment was performed with three repetitions. And the other culture mediums adopted in this study were the A1 medium [10.0 g soluble starch, 4.0 g yeast extract, 2.0 g peptone, 10.0 g CaCO_3_, 0.1 g KBr, and 0.04 g Fe_2_(SO_4_)_3_⋅4H_2_O], BF4 medium (4.0 g glucose, 4.0 g yeast extract, and 10.0 g malt extract), BF6 medium (20.0 g soluble starch, 0.1 g KNO_3_, 0.05 g K_2_HPO_4_, 0.05 g MgSO_4_⋅7H_2_O, 0.05 g NaCl, and 0.001 g FeSO_4_⋅7H_2_O), BF9 medium (10.0 g soluble starch, 1.0 g yeast extract, 1.0 g NaCl, 0.5 g KNO_3_, and 2.0 g CaCO_3_), and ZF1 medium (8 g soluble starch, 8 g soybean flour, 0.5 g K_2_HPO_4_, 0.34 g MgSO_4_⋅7H_2_O, 0.01 g FeSO_4_⋅7H_2_O, 0.13 g KNO_3_, and 0.5 g NaCl) dissolved in 1.0 L distilled water.

After fermentation of the strain, an equal amount of ethyl acetate was added and extracted three times. The organic phases were combined and concentrated under reduced pressure to obtain the crude extracts.

### Evaluation of antimicrobial activity

The plate confrontation method was performed for the evaluation of indicator strain antagonism. The indicator strains were incubated and cultured in a PDA medium at 28°C. A well-growth indicator strain cake (7 mm in diameter) was taken and placed in the center of the PDA plate. When the indicator strains grew to 1 cm in diameter, *Streptomyces* MDJKK11, MDJK44, and their co-culture spores were punctate incubated on both ends of the plate, and then continue to culture and record the antagonistic effect (Zhang et al., 2016).

The modified Paper Diffusion Method (Khan et al., 2019) and Microplate Spectrophotometer Method (Cox et al., 2010) for antimicrobial activity were performed for evaluation of the crude extract. In the modified Paper Diffusion Method, as for the indicator bacteria, 30 μL activated strain suspension per 40 mL medium was added and mixed into the cooled sterilized LB agar medium (about 55°C). Sterile filter paper pieces of 3–4 mm in diameter were added with 30 μL crude extract (10 mg crude extract dissolved in 1 mL methanol) per piece, 30 μL ciprofloxacin (CIP, 0.1 mg CIP dissolved in 1 mL methanol) for the positive control and 30 μL methanol for the negative control. The filter paper pieces evaporated the solvent and placed on the plate, and then incubated at 37°C. After 3–5 days, the antimicrobial activity was observed and recorded. As for the indicator fungi, the strains were spotted in the center of the PDA solid medium, and sterile filter paper pieces of about 3–4 mm in diameter were added with 30 μL of crude extract (10 mg crude extract dissolved in 1 mL methanol) per paper piece, 30 μL of positive control ketoconazole (0.1 mg ketoconazole dissolved in 1 mL methanol), 30 μL of negative control methanol, and then placed in the plates after evaporating the solvent. The plates were incubated at 28°C for 3–5 days to observe the effect of antimicrobial activity.

In the modified Microplate Spectrophotometer Method, as for the indicator bacteria, spores were inoculated in LB liquid medium and cultured for 12–24 h to give 1–2*10^6^ spores/mL suspension. 96-well plates were spiked with 100 μL suspension and 100 μL of LB medium per well. 50 μL LB medium, 50 μL crude extract, 50 μL methanol, and 50 μL ciprofloxacin (100 μg/mL dissolved in methanol) were added per well as the growth control, the sample, the negative control group, and the positive control, respectively. Three replicates of each treatment were incubated at 37°C for 12 h and then analyzed by a microplate reader at 630 nm. The inhibition rate of antimicrobial activity was evaluated as follows. Inhibition rate = [1–(OD_630 nm,48 h Treatment_−OD_630 nm,0 h Treatment_)/(OD_630 nm,48 h CK_−OD_630 nm,0 h CK_)]*100%. As for the indicator fungi, spores were inoculated into 200 mL mung beans medium and cultivated at 30°C and 180 r/min. After 3 days, 1–2*10^6^ spores/mL suspension was prepared with RPMI-1640 medium. 96-well plates were spiked with 100 μL spore suspension. 50 μL LB medium RPMI-1640 medium, 50 μL crude extract, 50 μL methanol, and 50 μL ketoconazole (100 μg/mL dissolved in methanol) were added per well as the growth control, the sample, the negative control group, and the positive control, respectively. Each treatment was carried out in triplicate. After incubated at 28°C for 1–2 days, the absorbance values were analyzed by a microplate reader at 260 nm. The inhibition rate of antimicrobial activity was evaluated as follows. Inhibition rate = [1−(OD_260 nm,48 h Treatment_−OD_260 nm,0 h Treatment_)/ (OD_260 nm,48 h CK_−OD_260 nm,0 h CK_)]*100%.

### Evaluation of growth-promoting effect by phosphorus and nitrogen dissolution test

As important essential elements in crop production, nitrogen, and phosphorus play a vital role in crop growth and development. The phosphorus dissolution test was performed as follows: MDJK11 and MDJK44 were incubated separately or together in organic [10.0 g glucose, 0.5 g (NH_4_)_2_SO_4_, 0.3 g MgSO_4_⋅7H_2_O, 0.03 g MnSO_4_⋅4H_2_O, 0.3 g KCl, 0.03 g FeSO_4_⋅7H_2_O, 0.3 g NaCl, 5.0 g CaCO_3_, 0.2 g lecithin and 20 g agar dissolved in 1.0 L distilled water] and inorganic phosphorus medium [10.0 g glucose, 0.5 g (NH_4_)_2_SO_4_, 0.3 g MgSO_4_⋅7H_2_O, 0.03 g MnSO_4_⋅4H_2_O, 0.3 g KCl, 0.03 g FeSO_4_⋅7H_2_O, 0.3 g NaCl, 10.0 g Ca_3_(PO_4_)_2_, and 20 g agar dissolved in 1.0 L distilled water], and then incubated at 28°C for 6 days. The colony growth and phosphorus solubilization and phosphorus dissolution circle were observed and recorded to evaluate. As for the nitrogen dissolution test, MDJK11 and MDJK44 were inoculated separately or together into a protein medium (3.0 g non-fat milk powder and 4.3 g agar dissolved in 0.2 L distilled water), and then incubated at 37°C for 2 d. The colony growth and nitrogen solubilization circle were observed and recorded to evaluate their nitrogen dissolution function (Cao et al., 2018).

### Evaluation of *Streptomyces* fermentation broth promoting wheat growth

A modified seed germination method (Nautiyal, 1999) was adopted to test the growth-promoting effect of MDJK11, MDJK44, and their co-culture fermentation on wheat (fermentation with BF4 medium at 30°C, 300 rpm shaker for 8 d). The fermentations were diluted with sterile water 10–400 times. The Shannong 27 wheat seeds were soaked with sterile water, 10, 50, 100, 200, and 400 times fermentation diluent, respectively. After 12 h, each treatment seeds were cultured at 27°C with a continuous corresponding processing solution. After being cultivated for 8 days, the root length and dry weight of the wheat were counted for statistical analyses.

### Determination of plant disease control effect of *Streptomyces*

The Detached-leaf method (Zhang et al., 2012) was adopted to assess the plant disease control effect of *Streptomyces*. Well-grown young leaves of wheat (trifoliate stage), maize (trifoliate stage), and tobacco were selected and cut to 5 cm in length, and then washed with deionized water. Each leaf was sprayed with 1 ml of *Streptomyces* fermentation crude extract (1 mg/ml dissolved in methanol), soaked and dried, and repeated three times. 2 μL (1 × 10^6^ CFU/mL) of *Fusarium graminearum*, *Fusarium capacity*, and *Fusarium acnes* spore suspensions were inoculated into the main veins of wheat, corn, and tobacco leaves to make them fully infested, respectively. The carbendazim (1 mg/mL dissolved in methanol) and methanol were set as the positive and negative control. The leaves were cultivated in a light incubator (24° 16h/20° 8 h with 60% humidity). After 5 days, the symptoms of wheat and corn leaves were observed and the spot size was measured. And the tobacco leaves were graded according to the standard (GB/T 17980.112-2004). The tobacco leaf disease grading index was as follows: Grade 0, no disease on the whole leaf; Grade 1, less than 2% of the leaf area infected; Grade 3, 3–10% of the leaf area infected; Grade 5, 11–20% of the leaf area infected; Grade 7, 21–50% of the leaf area infected; Grade 9, more than 51% of the leaf area infected. The disease and control index were calculated as follows. Disease index (%) = {Σ[(Number of diseased leaves at each level × Relative level value)]/(Total inoculum points surveyed × Top level representative value) × 100. Control rate (%) = (CK treatment disease index–Treatment disease index)/CK treatment disease index × 100}. Finally, as for wheat infected with *F. graminearum* and maize infected with *F. oxysporum*, the smaller the lesion diameter of the disease spot, the better the control effect. While for the tobacco infected with *F. moniliforme*, the larger the control rate, the better the control effect. And each treatment was performed at least three times.

### Metabonomic analysis based on Q Exactive UPLC-MS/MS

Thermo Scientific Q Exactive UPLC-MS/MS equipped with the Thermo Hypersil GOLD Q C18 column (2.1 × 100 mm, 1.9 μm) was adopted to analyze the acquired samples. The column temperature was set as 35°C and the flow rate was 0.3 mL/min. The mobile phase A was 0.1% acetic acid dissolved in water and B was 0.1% acetic acid dissolved in acetonitrile. And the gradient elution program was as follows: 0–0.5 min, 90% A; 0.5–7 min, 90% A; 7–8.5 min, 0% A; 8.6 min, 0–90% A; 8.6–10 min, 90% A. The injection volume was 3 μL. While the mass spectrometry method was as follows: the positive ion scan mode was set with 3.8 kV spray voltage, 40 sheath gas, 10 auxiliary gas, 350°C ion transport tube temperature, 17,500°C resolution, 1 microsleep number, 2*e^5^ AGC target, and 50 normalized collision energy. The negative ion mode was set with 2.9 kV spray voltage, 40 sheath gas, 0 auxiliary gas, 350°C ion transport tube temperature, 17,500°C resolution, 1 microsleep number, 2*e^5^ AGC target, and 50 normalized collision energy.

The data collected by Q Exactive UPLC-MS/MS were extracted and analyzed by Qualitative analysis software to analyze their primary and secondary mass spectra, and then further analyzed by Ms-Mine to compare the chromatograms of different samples and their main difference peaks; MS/MS public VS15 database matching was performed on the collected data using Ms-Dial Metabolite information. Five biological replicates of *Streptomyces* alone and co-culture were preprocessed on the MetaboAnalyst website for data to filter low-quality signal values and data with low reproducibility (reproducibility < 50%), and the missing data were processed for the lowest value (LOD*0.5), and the data were normalized and log10 transformed, and pareto scaling was processed to filter from Three groups of data with good repeatability were selected for subsequent analysis. Principal component analysis (PCA) and partial least squares discriminant analysis (PLS-DA) were used to analyze differences among groups (Zhou et al., 2018). The pairwise analysis was performed by orthogonal partial least squares analysis (OPLS-DA) to build the predictive model. And the Fold Change (FC > 2) and *P*-value (*p* < 0.001) were adopted to screen differential metabolites.

## Result and discussion

### *Streptomyces* antagonistic indicator strains and control of plant diseases

**FIGURE 2:**
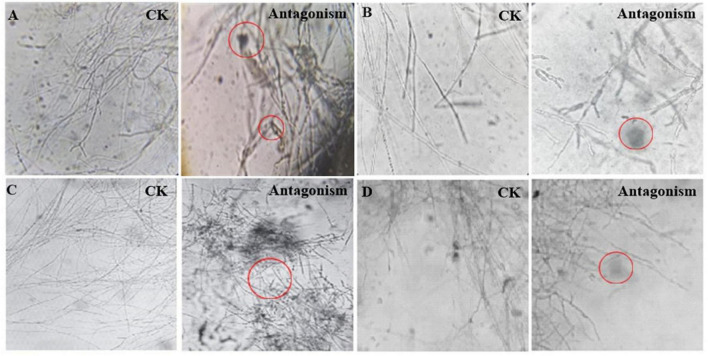
Microscopic morphology of *Streptomyces* MDJK11 and MDJK44 co-culture antagonistic indicator strains **(A)**, *Fusarium oxysporum*, **(B)**, *Fusarium solani*, **(C)**, *Fusarium moniliforme*, **(D)**, *Fusarium graminearum*, the red circles represent the abnormal phenomenon of the indicator strains).

*Streptomyces* MDJK44, MDJK11 monoculture, and co-culture showed a certain antagonistic effect on *F. oxysporum*, *F. solani*, *F. moniliforme*, and *F. graminearum*, among which the inhibitory effect on *F. oxysporum* and *F. graminearum* were better (Figure 2; Supplementary Figure 1 and Supplementary Table 1). It could be seen that the indicator fungus mycelia were full and intact state cultivated alone while changed to shrinkage, autolysis, and fracture under the inhibition of MDJK11 and MDJK44 co-culture in the microscope (Figure 2). Moreover, the effect of the co-culture condition was more obvious compared to the monoculture.

### Phosphorus and nitrogen solubilization and growth promotion of *Streptomyces*

**FIGURE 3:**
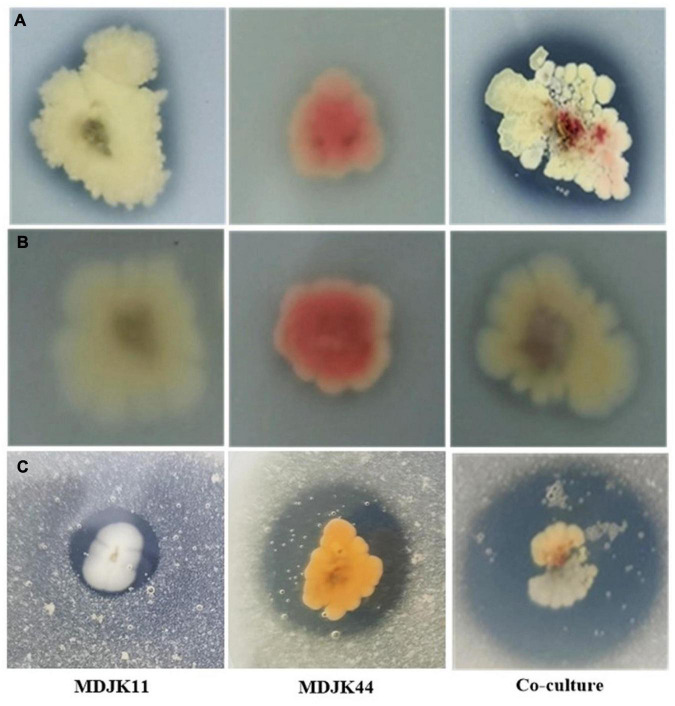
Phosphorus dissolution [**(A)**, inorganic phosphorus; **(B)**, organic phosphorus] and nitrogen decomposition **(C)** effects of *Streptomyces* MDJK11, MDJK44 monoculture, and co-culture.

*Streptomyces* MDJK44, MDJK11 monoculture, and co-culture could grow on organic and inorganic phosphorus medium, which indicated they could dissolve and utilize this phosphorus (Figure 3; Supplementary Table 2). The nitrogen solubilization test showed the nitrogen dissolving circle diameter was 15.61 ± 0.59, 9.95 ± 0.96, and 21.50 ± 2.59 mm for *Streptomyces* MDJK44, MDJK11 monoculture, and co-culture. It can be seen that co-culture significantly enhanced the effect of phosphorus and nitrogen dissolution (Figure 3; Supplementary Table 2).

### Metabonomics analysis of *Streptomyces* co-culture in different culture mediums

Microorganisms could produce different metabolites in various environments. To fully explore the metabolic potential of the *Streptomyces* co-culture, the One Strain Many Compounds (OSMAC) strategy was adopted to screen the fermentation medium. According to the pre-experiment, five culture mediums (A1, BF4, BF6, BF9, and ZF1) with different carbon and nitrogen sources were selected to further explore.

**FIGURE 4:**
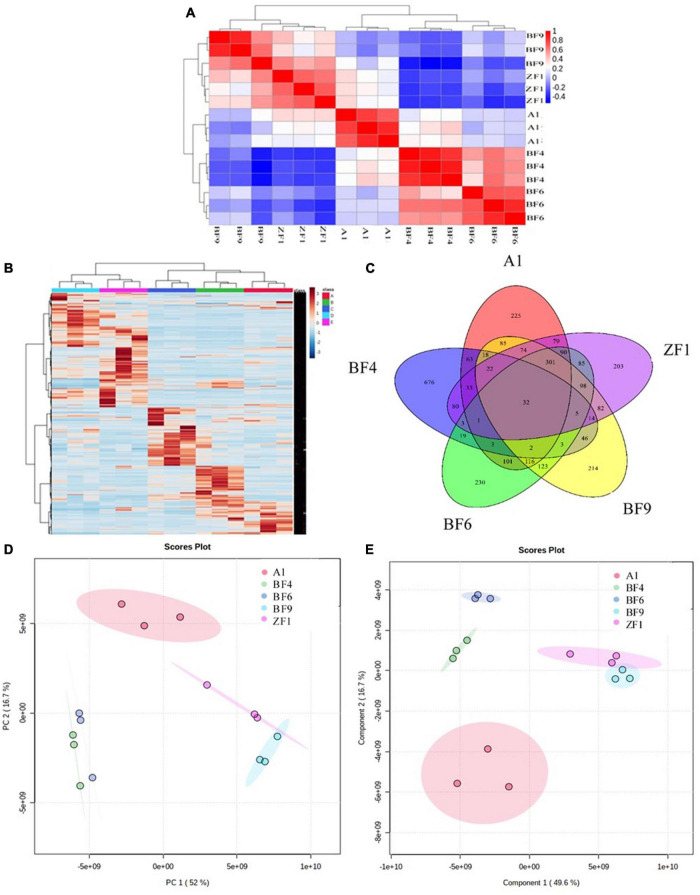
Metabonomic analysis of *Streptomyces* co-culture in five different culture mediums [**(A)**, correlation analysis; **(B)**, metabolite clustering analysis; **(C)**, Venn diagram; **(D)**, PCA analysis; and **(E)**, PLS-DA analysis].

To further screen the optimal conditions, the metabolites of five culture conditions were analyzed by LC-MS-based widely targeted metabonomics. The correlation analysis showed that the five conditions have well repeatability intragroup, which ensured the analysis accuracy (Figure 4A). As shown in Figure 4B, the clustering analysis showed that the metabolites of different conditions were well reproducible within groups and there were differences among groups. The Venn diagram showed that there were a total of 32 common metabolites between different culture conditions and 676 different metabolites in the BF4 medium compared with the other conditions among the analyzed metabolites (Figure 4C). And the numbers of different metabolites are 225, 204, 214, and 230 for A1, ZF1, BF9, and BF6 medium, respectively.

Multivariate statistical analysis of metabolites under different culture conditions also showed significant differences between groups. As shown in Figure 4D, PCA analysis revealed that there was 52% separation between groups on the first principal component (PCA1) and 16.7% separation on the second principal component (PCA2), and the differences between groups were more obvious on the PCA1. PLS-DA analysis showed that there was 49.6% separation on the PCA1 and 16.7% separation on the PCA2 (Figure 4E), which were consistent with the PCA analysis. For antifungal activity against *F. graminearum*, the inhibition rates were 98.9 ± 0.8%, 92.6 ± 5.4%, 74.3 ± 0.1%, 72.9 ± 3.8%, and 89.8 ± 1.3% for A1, BF4, BF6, BF9, and ZF1 fermentation crude extract (Supplementary Table 5). Based on the above test results, *Streptomyces* co-culture in BF4 medium condition had the advantage of high metabolic yield, high metabolic richness, and better antimicrobial activity compared with the other mediums and culture alone, which have great potential for exploration and application in plant growth promotion and disease prevention.

### Evaluation of the antimicrobial activities of crude extracts of *Streptomyces* monoculture and co-culture

The antimicrobial activities against different indicator strains of MDJK11, MDJK44 monoculture, and co-culture fermentation crude extracts (BF4 medium) were evaluated by the modified paper diffusion method and microplate spectrophotometer method. *F. acnes*, *F. cepacia*, *F. graminearum*, *F. solani*, *Escherichia coli* (gram-negative bacteria), and *Bacillus subtilis* (gam-positive bacteria, plant beneficial bacteria) were selected as the indicator strains.

For indicator pathogenic fungi, the inhibition rates (IR) against *F. graminearum* were 84.0 ± 1.4%, 89.7 ± 6.5%, and 92.6 ± 5.4% for MDJK44, MDJK11, and their co-culture (Supplementary Figure 2 and Supplementary Table 3). While they are 88.6 ± 1.2%, 92.3 ± 0.3%, and 94.3 ± 6.0% for *F. moniliforme* (Supplementary Figure 3 and Supplementary Table 3), 33.1 ± 9.9%, 32.1 ± 10.1%, and 75.7 ± 7% for *F. solani* (Supplementary Figure 4 and Supplementary Table 4), and 95.5 ± 0.8%, 98.4 ± 1.5%, and 90.2 ± 9.8% for *F. oxysporum* (Supplementary Figure 5 and Supplementary Table 4). MDJK11 and MDJK44 both had certain antibacterial activity against *B. subtilis*, and the inhibition rates (IR) were 97.3 ± 5.5% and 95.1 ± 5.0%, respectively, and it is noteworthy that the inhibition effect disappeared under the co-culture conditions (Supplementary Figure 6 and Supplementary Table 5). For *E. coli*, the co-culture condition showed relatively weak activity (83.7 ± 3.4% IR) than MDJK11 (99.3 ± 0.2% IR) and MDJK44 (85.7 ± 5.0% IR), but not as good as MDJK11 (Supplementary Figure 7 and Supplementary Table 5).

Based on the above results, as compared to MDJK11 and MDJK44 monocultures, the antifungal activities against *F. graminearum*, *F. solani*, and *F. oxysporum* were enhanced while there was no significant change against *F. moniliforme*. Meanwhile, the antibacterial activity against *E. coli* decreased, and the inhibitory effect on probiotic bacteria *B. subtilis* was significantly reduced or eliminated in the co-culture condition. It can be seen that the *Streptomyces* co-culture mode not only improves the antifungal activity against pathogenic fungi along with antifungal spectrum expansion, but also reduces the inhibition of probiotic bacteria.

### Evaluation and application of *Streptomyces* for growth promotion and antimicrobial activity

#### Exploration of *Streptomyces* co-culture for wheat growth promotion effect

**FIGURE 5:**
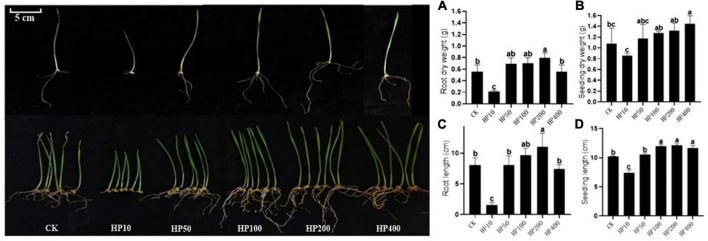
Effect of different dilutions of *Streptomyces* co-culture in BF4 medium fermentation broth on wheat growth promotion for 8 days [**(A)**, root dry weight; **(B)**, seedling dry weight; **(C)**, root length; **(D)**, seedling length; CK, treatment with water; HP10∼400, treatment with 10∼400 times fermentation broth dilution].

The crop wheat was selected to further evaluate the growth-promoting effect of *Streptomyces* co-culture in BF4 medium conditions. Results showed that the *Streptomyces* co-culture fermentation solution at 200 times dilution had obvious growth-promoting effects on wheat root and seedling (length and dry weight) as compared to the blank control. However, the 10 times dilution showed the opposite effect (Figure 5).

#### Study on the plant fungal diseases control of *Streptomyces* fermentation crude extracts

**FIGURE 6:**
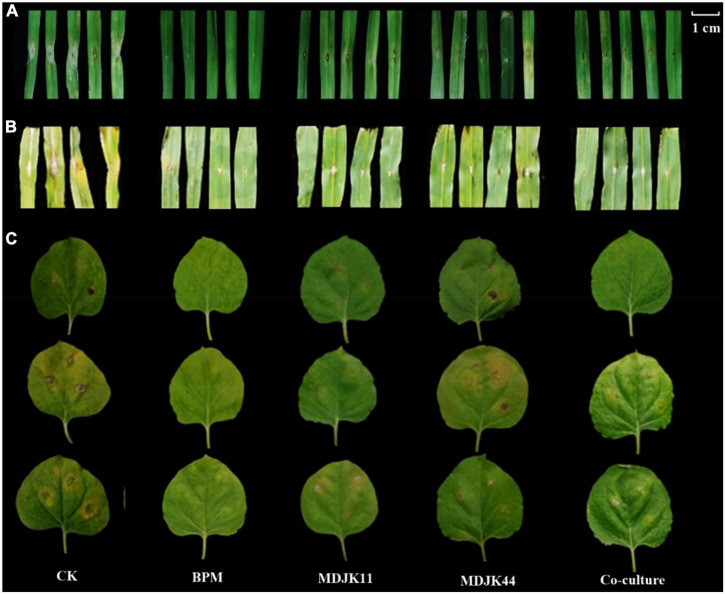
Effect of *Streptomyces* MDJK44, MDJK11 monoculture, and co-culture in BF4 medium fermentation broth crude extract on different plant diseases infected by pathogenic fungi for 5 days [**(A)**, wheat infected with *Fusarium graminearum*; **(B)**, maize infected with *Fusarium oxysporum*; **(C)**, tobacco infected with *Fusarium moniliforme*].

After *F. graminearum* infected the wheat leaves, the water-stained spots emerged at the inoculation site, the tips of the leaves turned yellow and shrinking, and dense mycelium was generated at the inoculation site (Figure 6). *Streptomyces* MDJK44, MDJK11 monoculture, and their co-culture fermentation crude extracts (BF4 medium, 1 mg dissolved in 1 mL methanol) showed good resistance against *F. graminis* compared with the control group and the disease spot diameters were 6.100, 5.464, 4.196, and 10.61 mm, respectively (Figure 6 and Table 1). Results showed the control effect of the co-culture condition was obviously better than that of MDJK44 and MDJK11 monoculture with a significant difference.

**TABLE 1:** Results of *Streptomyces* MDJK44, MDJK11 monoculture, and co-culture fermentation broth crude extract in BF4 medium on different plant diseases infected by pathogenic fungi.

Treatment	Wheat infected with *F. graminearum*	Maize infected with *F. oxysporum*	Tobacco infected with *F. moniliforme*	
	Lesion diameter (mm)	Lesion diameter (mm)	Disease index	Control rate (%)
CK	10.61 ± 1.35a	12.09 ± 3.13a	61.90 ± 3.10	−
BCM	1.52 ± 1.11d	2.65 ± 0.78c	0	100.00 ± 0.05a
MDJK44	6.10 ± 1.47b	5.24 ± 1.26bc	37.03 ± 1.85	39.74 ± 1.99c
MDJK11	5.46 ± 1.25bc	5.44 ± 1.71bc	40.70 ± 2.04	34.20 ± 1.71d
Co-culture	4.19 ± 0.88c	5.64 ± 2.33b	33.33 ± 1.67	46.20 ± 2.31b

As for *F. oxysporum*-infected maize leaves, black spots were formed on the infected part, dense hyphae were formed on the surface, and the middle part of the leaf shrunk with the whole leaf being de-greened, and the surface of the disease spot was covered with hyphae. On the contrary, if there is a certain resistance to *F. oxysporum*, there is no black plaque on the leaf surface, no or a small amount of hyphae, and almost no de-greening of the leaf. *Streptomyces* MDJK44, MDJK11 monoculture, and co-culture fermentation crude extracts all showed good resistance against *F. graminis* compared with the control group and the disease spot diameters were 5.24, 5.44, 5.64, and 12.09 mm, respectively. However, the control effects had no significant difference between the co-culture and monoculture and were all weaker than that of the positive control group (2.65 mm) (Figure 6 and Table 1).

As shown in Figure 6, after 5 days of *F. moniliforme*-infected tobacco leaves, the blank control treatment showed large anthracnose spots with more pathogenic mycelium accompanied by the leaves’ wrinkling and green-loss. And the disease index was 61.90 ± 3.10 after correlation calculations. On the contrary, the positive control treatment obviously had a strong inhibitory effect against *F. acnes* infestation with no shrinkage and green-loss symptoms, and almost no disease spots produced. And the disease index and control effect were 0 and 100 ± 0.05%, respectively. *Streptomyces* MDJK44, MDJK11 monoculture, and co-culture fermentation crude extracts all showed good resistance against *F. acuminatum* compared with the control group. The disease index and control effect were 37.03 ± 1.85 and 39.74 ± 1.99%, 40.70 ± 2.04 and 34.20 ± 1.71%, 33.33 ± 1.67, and 46.20 ± 2.31% (Figure 6 and Table 1).

### Analysis of the material basis of *Streptomyces* promotion and disease prevention under different culture conditions based on metabonomics

#### Correlation analysis of metabolite base

To further clarify the material basis of plant promotion and disease prevention in *Streptomyces* MDJK44, MDJK11 monoculture, and co-culture conditions, and elucidate the advantages of co-culture, LC-MS based metabonomics was adopted to analyze their fermentation crude extracts.

**FIGURE 7:**
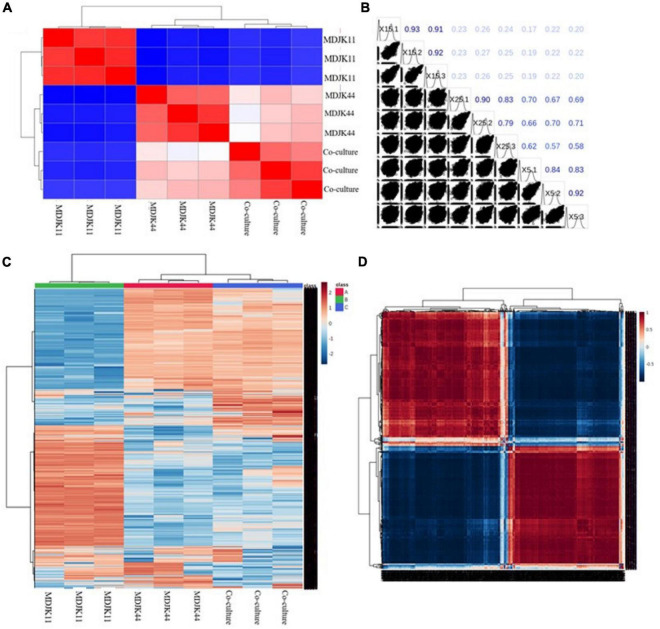
Metabonomic analysis of *Streptomyces* MDJK44, MDJK11 monoculture, and co-culture in BF4 medium [**(A)**, correlation plot; **(B)**, correlation matrix plot; **(C)**, cluster analysis plot; and **(D)**, correlation heat map].

Both the correlation analysis with spearman function (intra-group correlation > 0.8, Figures 7A,B) and the clustering analysis of metabolites (Figures 7C,D) showed each condition had good biological replicates intra group with good homogeneity and data reliability. The clustering analysis also further demonstrated that the co-culture had significant intergroup differences with the monoculture, which significantly increased the metabolite species and content to some extent and there were multiple differential metabolites to be further explored (Figures 7C,D).

**FIGURE 8:**
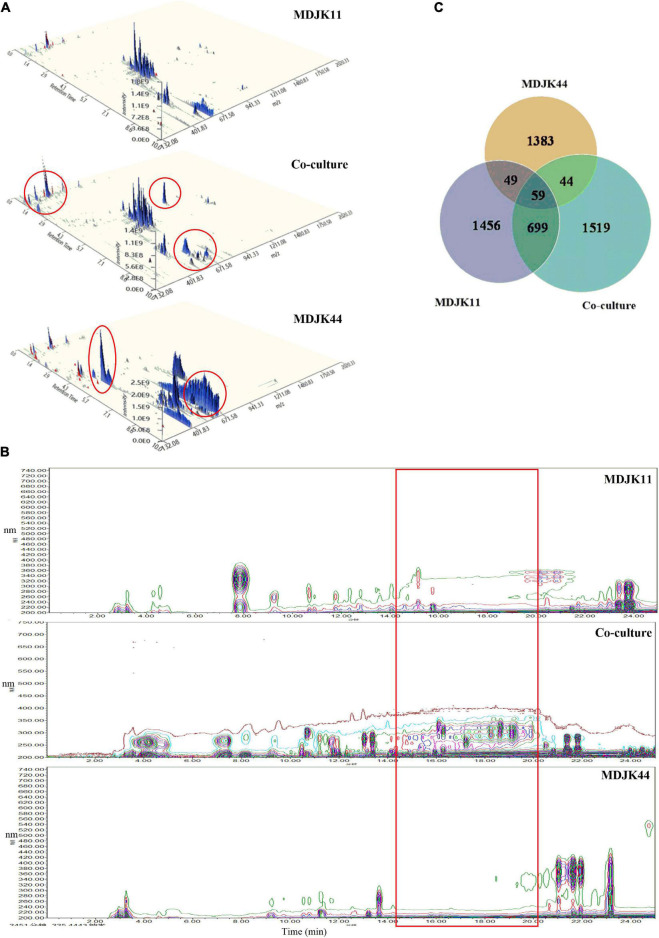
The whole-ion scans **(A)**, HPLC fingerprint profiles **(B)**, and statistical Venn diagram of the identified metabolites **(C)** of *Streptomyces* MDJK11 and MDJK44 in monoculture and co-culture (the red circles represent the metabolic differences).

The full ion scan of metabolites (Figure 8A) showed the co-culture condition was significantly different from that of the monoculture conditions, which was more abundant with differential metabolite ion peaks accompanied by some ion peaks disappearing (marked with red circles).

#### Analysis of differential metabolites inter groups

*Streptomyces* MDJK44, MDJK11 monoculture, and co-culture conditions had certain growth promoting and antimicrobial activity with certain differences.

To further clarify the material basis, the acquired LC-MS data were matched by the MS/MS public VS15 database. A total of 2,262, 1,535, and 2,321 metabolites were identified for MDJK11, MDJK44, and the co-culture conditions, respectively. The Venn diagram showed that 1,519 metabolites were changed or newly increased in the co-culture condition, and the metabolites shared with MDJK11 and MDJK44 were 758 and 103 species, respectively (Figure 8B).

The metabolites were classified according to their chemical structure characteristics and functional activities, mainly including probiotics and antimicrobics. The probiotics included Phytohormones, Coumarins, and Phenolic acid metabolites while the antimicrobics included Aminoglycosides, Amines, Macrolides, Peptides, Terpenoids, Alkaloids, Steroids, and Volatile Organic Compounds (VOCs) metabolites. Compared to MDJK44 and MDJK11 monoculture, the co-culture significantly increased the abundance of terpenoids, alkaloids, steroids, macrolides, and peptides (Table 2). Further analysis showed that organic acids were significantly increased in species and alkaloids, amino acids, glycosides, and terpenoids were significantly increased in content in co-culture conditions. On the contrary, desertomycins with broad-spectrum antibacterial activity disappeared significantly.

**TABLE 2:** Statistics and classification of the identified metabolites in *Streptomyces* MDJK11 and MDJK44 in monoculture and co-culture.

Type	MDJK44	MDJK11	Co-culture
Phytohormones	7	13	10
Aminoglycosides	5	9	8
Amines	44	57	55
Macrolides	23	28	35
Peptides	71	67	70
Terpenoids	193	232	264
Alkaloids	290	384	355
Steroids	60	63	71
Coumarins	42	56	51
Phenolic acid	14	17	18
Others	786	1336	1384
Total	1535	2262	2321

#### Multivariate statistical analysis of inter-group differences

**FIGURE 9:**
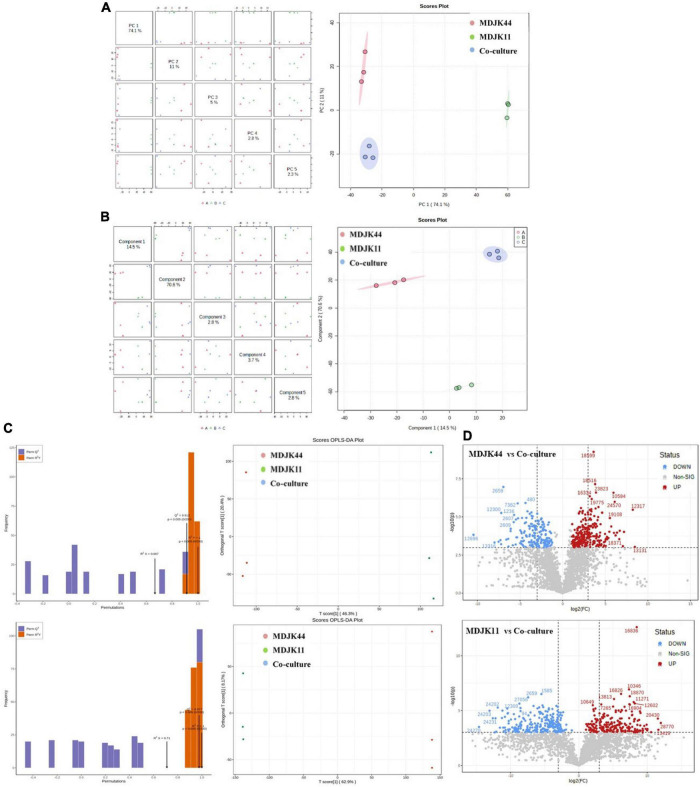
Multivariate statistical analysis of *Streptomyces* MDJK11 and MDJK44 in monoculture and co-culture [**(A)**, PCA scores graphs; **(B)**, PLS-DA graphs; **(C)**, Scores OPLS-DA plots; and **(D)**, differential metabolite volcano graphs].

The PCA analysis can reflect metabonomics characteristics under multidimensional data by different principal components, thus the differences inter groups can be easily observed. Herein, the PCA results showed obvious separation of different culture modes, which are consistent with the analyzed metabolite differences above. The first principal component (PC1) shown in Figure 9A can explain 74.1% of the original dataset characteristics, where the difference between MDJK11 monoculture and co-culture can be found. The second principal component (PC2) can explain 11% of the original dataset characteristics, and the difference between MDJK44 monoculture and co-culture can be found in PC2. And the PC3∼PC5 principal components can explain 5%, 2.8%, and 2.3% of the features, which indicate that the two *Streptomyces* had characteristic metabolic profiles in monoculture and co-cultures, respectively.

Partial least squares discriminant analysis (PLSDA) is similar to the PCA, which separates the groups as much as possible by appropriate rotation adjustment of the principal component space. To improve the metabonomic approach to identify the differences inter groups, a statistical prediction model was used to predict the metabolite profiles under different groups. As shown in Figure 9B, PC1 could explain 14.5% of the original dataset characteristics, which could explain the separation degree between MDJK44 and co-culture. And the PC2 could explain 70.6% of the characteristics of the original dataset, which could explain the difference between MDJK11 and co-culture. Both the PLS-DA and the PCA analysis showed that the co-culture condition could significantly change the metabolic spectrum of *Streptomyces* compared with the monoculture.

The OPLS-DA analysis is a multivariate statistical method for supervised pattern recognition, which can effectively screen for differential metabolites by eliminating the irrelevant effects. The score maps were created by the OPLS-DA to analyze the metabolic components of MDJK44, MDJK11, and the co-culture separately (Figure 9C). In this model, R2X and R2Y represent the explanation rate of the X and Y matrices, respectively, and Q2 represents the prediction ability. The Q2 values of MDJK44 and MDJK11 compared with the co-culture were 0.913 and 0.977, respectively. Both of the Q2 values were greater than 0.9, which indicates the proposed model was very ideal (Figure 9C). The OPLS-DA score plots showed that different comparison groups had significant separation, which indicates that the metabolites of MDJK44, MDJK11, and the co-culture *Streptomyces* had significant difference.

Fold Change (FC) and *P*-value were further adopted to screen the differential metabolites with the screening criteria of FC > 2 and *p* < 0.001 as targeted metabolites. Results showed that there were 252 upregulated metabolites and 178 downregulated metabolites in the comparison of MDJK44 and co-culture, among which the upregulated products included 27 alkaloids, 25 steroids, 15 peptides, 12 macrolides, 2 aminoglycosides, 1 phytohormone, and 170 other substances (Figure 9D). And the differential metabolites number was 264 in the comparison of MDJK11 and co-culture, among which the upregulated metabolites included 33 alkaloids, 29 peptides, 10 steroids, 2 macrolides, 2 phytohormones, 1 aminoglycoside, and 187 others (Figure 9D).

#### Analysis of probiotic and antimicrobial metabolites and their differences in monoculture and co-culture condition

In total, *Streptomyces* MDJK44, MDJK11 monoculture, and their co-culture conditions have significant differences in metabolites. To further clarify the material basis of probiotics and disease prevention, the classification discussion was conducted as follows.

#### Probiotic metabolites

**FIGURE 10:**
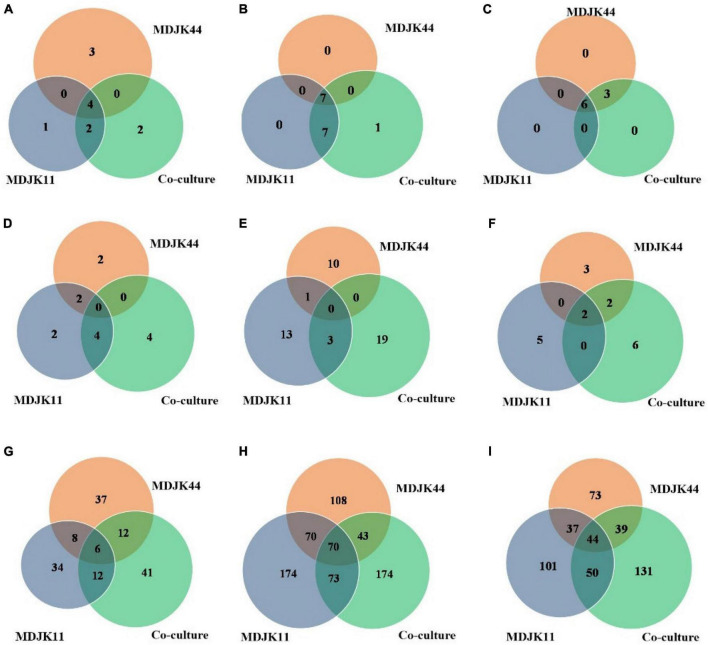
Statistical Venn diagrams for different identified metabolites of *Streptomyces* MDJK11 and MDJK44 in monoculture and co-culture [**(A)**, plant hormones; **(B)**, coumarin; **(C)**, phenolic acid; **(D)**, aminoglycosides; **(E)**, macrolides; **(F)**, cyclic peptides; **(G)**, alkaloid; **(H)**, terpenoids; and **(I)**, Steroids].

Metabonomic analysis showed that phytohormones, coumarins, and phenolic acid metabolites can act as probiotic substances in *Streptomyces* MDJK44, MDJK11 monoculture, and their co-culture conditions. Phytohormones as natural products with probiotic function, as shown in Figure 10, there were seven cytokinins, four salicylates, three jasmonates, one abscisic acid, and one gibberellin under different cultures. A total of four types of phytohormones were detected in the three cultivate models and abscisic acid substances were identified in the co-culture condition, which were not detected under *Streptomyces* MDJK44 and MDJK11 monoculture conditions (Figure 10).

The phytohormones detected under different culture conditions provide a scientific basis for explaining their growth-promoting effects. The results indicated that the co-culture could change or inhibit the biosynthesis pathway of certain metabolites, such as inhibiting the production of jasmonic acid while promoting abscisic acid. As shown in the wheat growth promotion test, the growth promotion effect of a low concentration of fermentation solution was better than that of a high concentration, which was presumed to be caused by the duality of gibberellin or growth hormone in phytohormones (Costacurta and Vanderleyden, 1995).

Coumarins have antimicrobial activity, cell protection, and plant growth promotion by eliminating the reactive oxygen (Kostova et al., 2011). A total of 15 coumarins were detected in *Streptomyces* and their co-culture conditions, pyranocoumarins, 4-methylcoumarin, carotenoid coumarin, hydroxycoumarin, and psoralen included. A total of seven metabolites in the three cultivate models were detected and the co-culture condition increased the number of species as compared to the monoculture.

Phenolic acids as plant growth promotion, stress resistance, signaling molecules, and chemosensory substances are attracting more and more attention (Kumar and Goel, 2019). A total of nine phenolic acids were detected in this study, including *p*-methoxybenzoic acid, *m*-methoxybenzoic acid, hydroxybenzoic acid, cinnamic acid esters, and cinnamic acid esters. There were six metabolites in three culture conditions and the co-culture increased the metabolites relative to MDJK44 monoculture.

#### Antimicrobial and disease prevention metabolites

For antimicrobial and disease prevention metabolites, antibiotics play an important role as a kind of indispensable secondary metabolites. The co-culture condition not only stimulated the production not produced in the monoculture, but also increased the content of substances already produced in the monoculture (Figure 10). The detailed analysis and discussion are as follows.

Aminoglycosides, as common metabolites of *Streptomyces*, are often utilized as Gram-negative bacteria antibiotics of preferred choice (Krause et al., 2016). A total of 12 aminoglycosides were identified in *Streptomyces* MDJK44, MDJK11 monoculture, and their co-culture conditions. Moreover, the co-culture condition produced four components that were not yet detected in *Streptomyces* MDJK44 and MDJK11 monoculture (Figure 10). The results may explain *Streptomyces* MDJK44 and MDJK11 monoculture showed better inhibition activity against *E. coli*, while the inhibition effect decreased significantly after co-culture, which presumably due to the inhibition of certain anti-Gram-negative substance and provided references for further study.

Macrolide metabolites, as one of the common natural antibiotics, could inhibit both gram-negative and positive bacteria with broad-spectrum (Vázquez-Laslop and Mankin, 2018). A total of 47 macrolides were identified under the three culture conditions, of which 11 were produced by MDJK44, 16 by MDJK11, and 23 by the co-culture condition (Figure 10). The co-culture significantly increased the abundance of macrolides, which provide material bases for their good antimicrobial activity.

**FIGURE 11:**
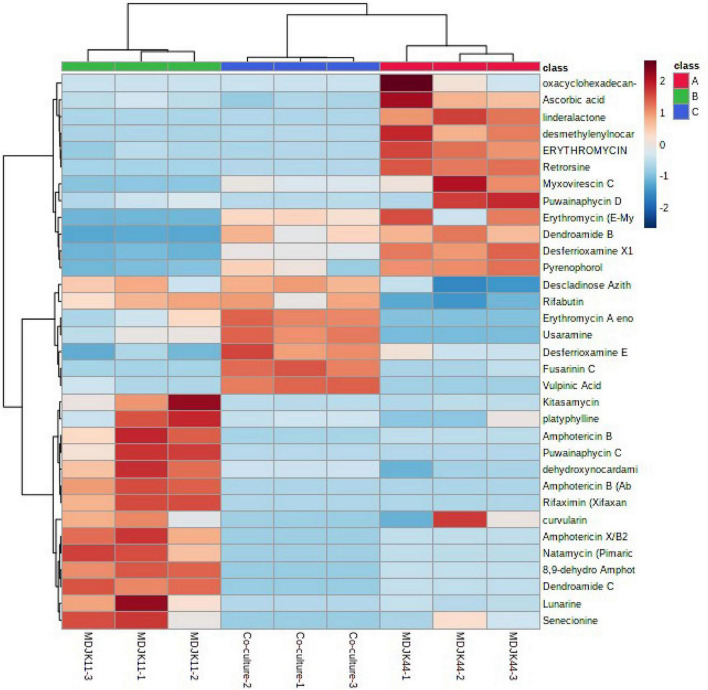
Heat map of the identified antibiotics of *Streptomyces* MDJK11 and MDJK44 in monoculture and co-culture.

Cyclic peptides have the characteristics of high stability, special structure, rich activity, and wide distribution. The antimicrobial mechanism of most cyclic peptides is similar, that is they can enter the cell plasma membrane to interfere with the cell plasma membrane barrier by self-promotion. Peptide antibiotics do not easily cause resistance and are not prone to cross-resistance with other antibiotics, which attract continuous attention (Lacey and Rutledge, 2022). A total of 17 cyclic peptides were identified under the three culture conditions, of which seven were produced by MDJK44, 7 by MDJK11, and 10 by the co-culture condition (Figure 10). The co-culture not only stimulated the production of new substances which were not detected in co-culture condition, but also increased some peptides’ relative yield abundance (Figure 11).

Steroids, terpenoids, and alkaloids have antimicrobial, anti-adversity, and antioxidant effects (Fernandes et al., 2003; Xu et al., 2014; Kuzuyama, 2017). Metabonomic analysis showed that a total of 194 steroids were identified under the three culture conditions, of which 60 were produced by MDJK44, 63 by MDJK11, and 71 by the co-culture (Figure 10). The co-culture increased the abundance of steroids to a certain extent, of which 41 were differential components compared with MDJK11 and MDJK44 monoculture. As for terpenoids, a total of 689 terpenoids were identified under the three culture conditions, of which 193 were produced by MDJK44, 232 by MDJK11, and 264 by the co-culture conditions (Figure 10). The co-culture condition could obviously increase the abundance of steroids to a certain extent, among which there were 131 differential components compared with MDJK11 and MDJK44 monoculture. As for alkaloid metabolites, a total of 712 alkaloids were analyzed and identified under the three culture conditions, of which 291 were produced by MDJK44, 390 by MDJK11, and 360 by the co-culture conditions (Figure 10). The co-culture significantly increased the abundance of steroids to a certain extent, of which 131 were differential components compared with MDJK11 and MDJK44 monoculture.

Moreover, in addition to the identified metabolites based on metabonomics in *Streptomyces* MDJK44, MDJK11 monoculture, and their co-culture conditions, there are still a large number of novel unidentified metabolites, which need further exploration, and continuous research is very worthy and valuable.

In conclusion, the co-culture of the two *Streptomyces* could effectively play a synergistic role, and the growth promotion and antimicrobial effects had been enhanced. At the level of metabolites, there were certain differences in the types and contents of metabolites between co-culture and monoculture. We speculated that the possible mechanism behind this is that on the one hand co-culture can simulate the growth and nutritional competition in the natural environment to a certain extent, on the other hand, it may be that the co-culture model could induce the activation and expression of some silenced genes as previous reports (Marmann et al., 2014; Maglangit et al., 2020). And the speculations need further exploration and verification.

## Conclusion

The study showed that the co-culture of *Streptomyces albireticuli* MDJK11 and *Streptomyces albofavus* MDJK44 had a synergistic effect compared with the monoculture, which could enhance the function of dissolving phosphorus and nitrogen and promoting the growth of wheat roots and seedlings. Moreover, the co-culture also has a good control effect on wheat, corn, and tobacco diseases infected by pathogenic fungi. Based on metabonomics analysis, the material basis of growth promotion and disease prevention of *Streptomyces* were plant hormones and antibiotics, respectively. And co-culture conditions can significantly induce *Streptomyces* to produce a more abundant and higher content of the above substances. The research provides a scientific basis for *Streptomyces* to be used in plant growth promotion and disease prevention and further provides references for microbial co-culture mode to achieve synergy.

## Data availability statement

The raw data supporting the conclusions of this article will be made available by the authors, without undue reservation.

## Author contributions

JL performed the plant disease control experiments and wrote the original manuscript. LZ performed the metabonomic analysis. LXZ guided and supervised the experiments. GY screened the fermentation medium. JLL performed the strain fermentation. CW, XM, and YD checked the manuscript. BD provided the experimental strains and supervised the experiments. XM guided and supervised the experimental scheme and provide funding support. All authors contributed to the article and approved the submitted version.
