# Supplementary material for: Synergistic effect of co-culture rhizosphere Streptomyces: A promising strategy to enhance antimicrobial activity and plant growth-promoting function
Source: Front Microbiol. 2022 Aug 11;13:976484. doi: 10.3389/fmicb.2022.976484 (PMC9403869; doi:10.3389/fmicb.2022.976484)
Supplement: Supplementary file 1 [file Data_Sheet_1.docx]

Supplementary Material

Synergistic Effect of Co-culture Rhizosphere *Streptomyces* : A Promising Strategy to Enhance Antimicrobial Activity and Plant Growth-Promoting Function

Jing Li^1,§^, Lin Zhang^1,§^, Gan Yao^2^, Lixiang Zhu^1^, Jingling Lin^1^, Chengqiang Wang^2^, Binghai Du^2^, Yanqin Ding^2,*^, Xiangui Mei^1,*^

^1^State Key Laboratory of Crop Biology, College of Agronomy, Shandong Agricultural University, Taian, Shandong 271018, China

^2^College of life sciences, Shandong Agricultural University, Taian, Shandong 271018, China;

*** Correspondence:**Corresponding Author
Xiangui Mei (E-mail: meixiangui@163.com), Yanqin Ding (E-mail: dyq@sdau.edu.cn)

**^§^**These authors contributed equally to this work.

# Supplementary Figures and Tables

## Supplementary Figures


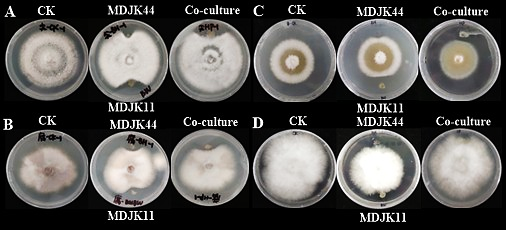


**FIG S1** *Streptomyces* MDJK11, MDJK44 mono-culture and co-culture antagonistic indicator strains (A, *F. oxysporum*, B, *F. solani*, C, *F. moniliforme*, D, *F. graminearum*)


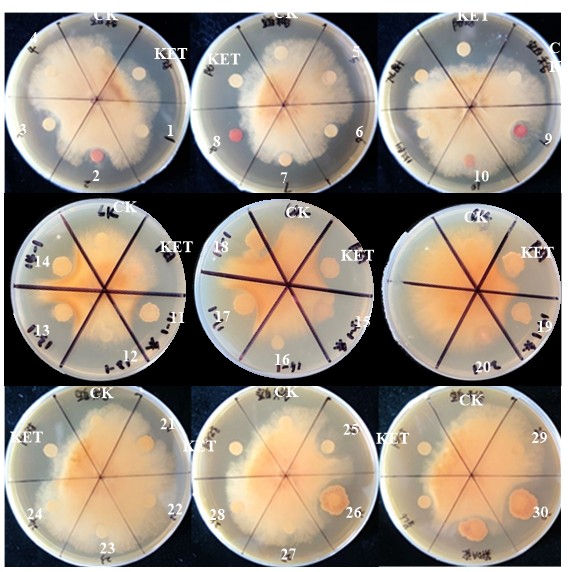


**FIG S2** Inhibition of *Streptomyces* fermentation crude extract against *F.* graminearum (1-30 represent *Streptomyces* MDJK11, MDJK44 mono-culture and co-culture conditions in the F2, A1, BF1, BF3, BF4, BF5, BF6, BF9, ZF1 and ZF2 culture medium, respectively)


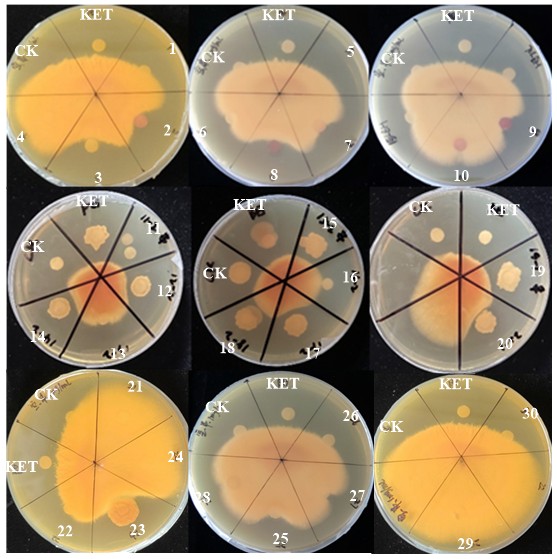


**FIG S3** Inhibition of *Streptomyces* fermentation crude extract against *F. moniliforme* (1-30 represent *Streptomyces* MDJK11, MDJK44 mono-culture and co-culture conditions in the F2, A1, BF1, BF3, BF4, BF5, BF6, BF9, ZF1 and ZF2 culture medium, respectively)


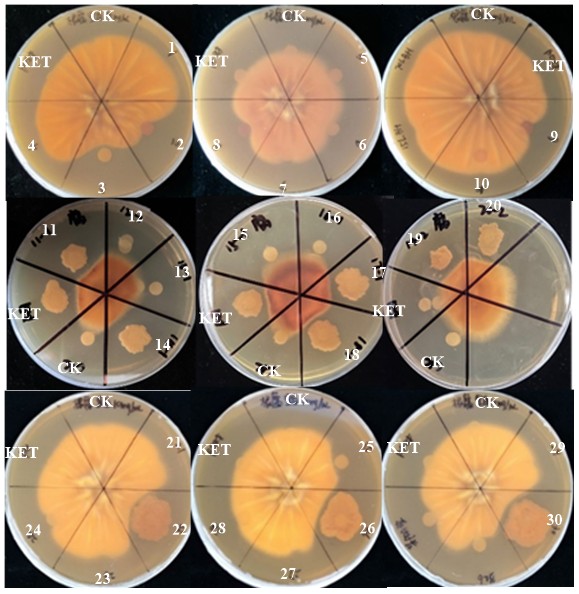


**FIG S4** Inhibition of *Streptomyces* fermentation crude extract against *F. solani* (1-30 represent *Streptomyces* MDJK11, MDJK44 mono-culture and co-culture conditions in the F2, A1, BF1, BF3, BF4, BF5, BF6, BF9, ZF1 and ZF2 culture medium, respectively)


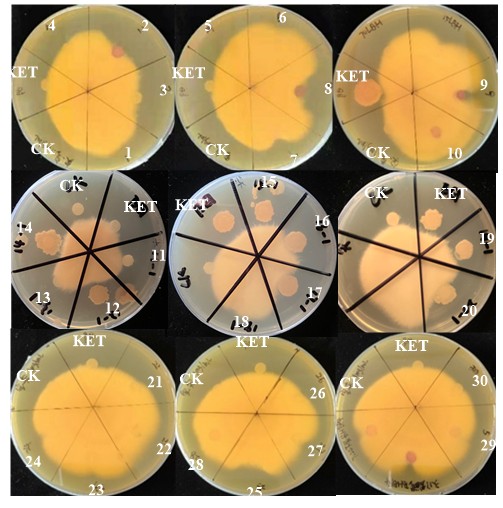


**FIG S5** Inhibition of *Streptomyces* fermentation crude extract against *F. oxysporum* (1-30 represent *Streptomyces* MDJK11, MDJK44 mono-culture and co-culture conditions in the F2, A1, BF1, BF3, BF4, BF5, BF6, BF9, ZF1 and ZF2 culture medium, respectively)


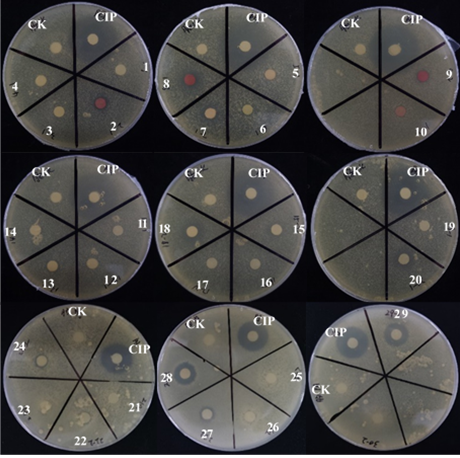
.

**FIG** **S6** Inhibition of *Streptomyces* fermentation crude extract against *B. subtilis* (1-30 represent *Streptomyces* MDJK11, MDJK44 mono-culture and co-culture conditions in the F2, A1, BF1, BF3, BF4, BF5, BF6, BF9, ZF1 and ZF2 culture medium, respectively)


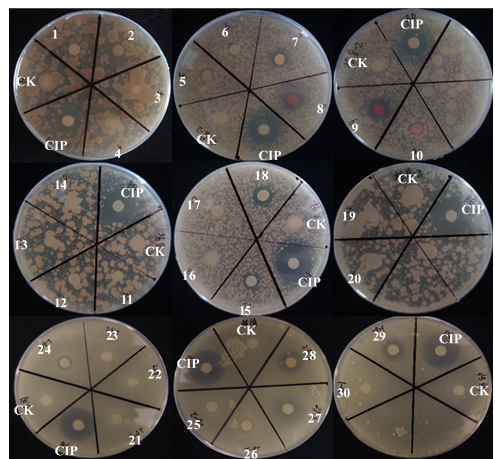


**FIG S7** Inhibition of *Streptomyces* fermentation crude extract against *E. coli* (1-30 represent *Streptomyces* MDJK11, MDJK44 mono-culture and co-culture conditions in the F2, A1, BF1, BF3, BF4, BF5, BF6, BF9, ZF1 and ZF2 culture medium, respectively)


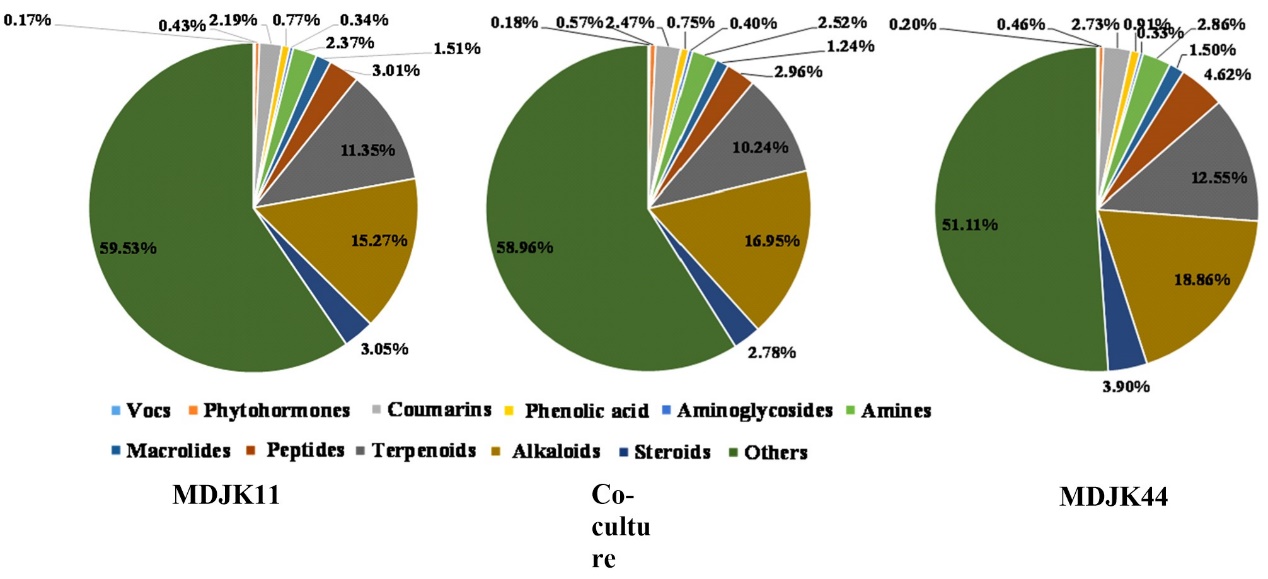


**FIG S8** Statistics and classification pie chart of the identified metabolites in *Streptomyces* MDJK11 and MDJK44 in mono-culture and co-culture

## Supplementary Tables

**Table S1** The inhibition rates of *Streptomyces* MDJK11, MDJK44 mono-culture and co-culture antagonistic indicator strains

| Indicator strains | Inhibition rates | | |
| --- | --- | --- | --- |
|  | MDJK11 | MDJK44 | Co-culture |
| *F. oxysporum* | 31.63%±1.94% | 34.35%±1.25% | 41.68%±6.38% |
| *F. solani* | 27.83%±3.76% | 26.90%±3.23% | 30.31%±2.14% |
| *F. moniliforme* | 39.55%±2.33% | 38.21%±0.55% | 32.61%±7.79% |
| *F. graminearum* | 26.77%±1.91% | 23.30%±3.02% | 39.63%±4.58% |

**Table S2** The results of phosphorus dissolution and nitrogen decomposition effects of *Streptomyces* MDJK11, MDJK44 mono-culture and co-culture

| Strain |  | MDJK11 | MDJK44 | Co-culture |
| --- | --- | --- | --- | --- |
| Inorganic phosphorus | D (mm) | 11.04±0.56 | 0 | 13.37±0.64 |
|  | d (mm) | 9.91±0.55 | 0 | 10.89±0.17 |
|  | D/d | 1.11±0.01 | 0 | 1.23±0.07 |
| Organic phosphorus | D (mm) | 0 | 10.50±1.40 | 13.00±0.65 |
|  | d (mm) | 0 | 9.37±1.48 | 11.63±1.03 |
|  | D/d | 0 | 1.12±0.03 | 1.12±0.04 |
| Nitrogen decomposition | D (mm) | 9.95±0.96 | 15.61±0.59 | 21.50±2.59 |
|  | d (mm) | 6.83±1.41 | 9.38±1.25 | 6.05±1.12 |
|  | D/d | 1.45±0.23 | 1.66±0.22 | 3.54±0.32 |

Note : D, diameter of dissolving circle; d, strain growth diameter.

**Table S3** The antifungal inhibition rates of *Streptomyces* MDJK11, MDJK44 mono-culture and co-culture against *F. moniliforme* and *F.* graminearum in different culture media

| Culture medium | Inhibition Rate (%) | | | | | |
| --- | --- | --- | --- | --- | --- | --- |
|  | *F. moniliforme* | | | *F.* graminearum | | |
|  | MDJK44 | MDJK11 | Co-culture | MDJK44 | MDJK11 | Co-culture |
| F2 | 11.0±3.2 | 94.0±6.0 | 94.8±4.5 | 97.4±0.1 | 97.8±0.9 | 91.4±7.0 |
| A1 | 95.3±3.3 | 92.8±5.0 | 96.5±3.0 | 99.7±2.9 | 96.6±0.5 | 98.9±0.8 |
| BF1 | 94.5±3.1 | 98.4±0.5 | 98.1±1.4 | 95.5±7.1 | 99.4±0.2 | -33.7±5.7 |
| BF3 | 97.2±2.8 | 96.1±3.0 | 88.8±1.7 | 98.6±1.2 | 95.8±4.6 | -72.5±16.4 |
| BF4 | 88.6±1.2 | 92.3±0.3 | 94.3±6.0 | 84.0±1.4 | 89.7±6.5 | 92.6±5.4 |
| BF5 | 92.0±1.4 | 76.8±2.3 | -53.6±21.7 | 97.6±1.7 | 94.1±9.6 | 56.6±2.2 |
| BF6 | 86.8±2.2 | 95.1±1.8 | 92.6±6.5 | 79.5±0.9 | 97.8±0.6 | 74.3±0.1 |
| BF9 | 92.7±1.9 | 85.5±7.9 | 87.9±4. | 99.0±2.6 | 86.6±0.1 | 72.9±3.8 |
| ZF1 | 91.9±1.0 | 80.5±3.2 | 90.3±1.2 | 93.9±3.5 | 91.7±0.0 | 89.8±1.3 |
| ZF2 | 70.6±5.1 | 95.0±1.4 | 72.4±6.3 | 97.6±5.8 | 97.0±0.4 | 13.6±0.1 |
| Growth control | -88.2±18.1 | | | -222.9±1.3 | | |
| Negative control | 0 | | | 0 | | |
| Positive control | 96.0±3.5 | | | 85.9±6.1 | | |

**Table S4** The antifungal inhibition rates of *Streptomyces* MDJK11, MDJK44 mono-culture and co-culture against *F. oxysporum* and *F. solani* in different culture media

| Culture medium | Inhibition Rate (%) | | | | | |
| --- | --- | --- | --- | --- | --- | --- |
|  | *F. oxysporum* | | | *F. solani* | | |
|  | MDJK44 | MDJK11 | Co-culture | MDJK44 | MDJK11 | Co-culture |
| F2 | 50.1±11.9 | 75.2±6.5 | 94.5±6.4 | 89.1±10.7 | 62.3±11.3 | 44.6±0.7 |
| A1 | 98.2±0.9 | 60.3±8.0 | 98.8±0.6 | 97.1±1.1 | 86.9±0.9 | 90.7±0.2 |
| BF1 | 96.8±0.8 | 80.7±4.0 | 97.8±0.5 | 94.7±0.9 | 95.6±4.7 | 60.4±1.7 |
| BF3 | 99.3±1.8 | 41.4±5.4 | 97.7±0.7 | 85.3±6.8 | 99.9±1.8 | 73.9±1.4 |
| BF4 | 33.1±9.9 | 32.1±10.1 | 75.7±7.0 | 98.4±1.5 | 95.5±0.8 | 90.2±7.2 |
| BF5 | 99.1±1.4 | -4.6±12.9 | 18.0±2.2 | 73.0±22.0 | 85.1±11.4 | -128.3±4.7 |
| BF6 | 96.6±1.9 | -23.1±2.7 | 98.2±0.7 | 73.7±5.2 | 69.9±1.3 | 92.3±3.9 |
| BF9 | 95.1±4.5 | 67.6±5.5 | 83.2±1.0 | 94.3±2.0 | 98.5±0.2 | 85.7±6.2 |
| ZF1 | 94.5±1.4 | 69.8±7.5 | 86.1±7.8 | 98.8±1.1 | 83.5±2.6 | 92.9±5.2 |
| ZF2 | 94.8±5.7 | 77.0±7.6 | 99.9±0.0 | 82.9±4.9 | 93.0±2.5 | -130.0±4.8 |
| Growth control | -46.9±1.1 | | | -139.6±5.6 | | |
| Negative control | 0 | | | 0 | | |
| Positive control | -46.9±1.1 | | | -139.6±5.6 | | |

**Table S5** The antibacterial inhibition rates of *Streptomyces* MDJK11, MDJK44 mono-culture and co-culture against indicator bacteria in different culture media

| Culture medium | Inhibition Rate (%) | | | | | |
| --- | --- | --- | --- | --- | --- | --- |
|  | *E. Coli* | | | *B. Subtilis* | | |
|  | MDJK44 | MDJK11 | Co-culture | MDJK44 | MDJK11 | Co-culture |
| F2 | 63.7±0.8 | 72.0±2.0 | 0.90±0.2 | 99.7±0.2 | 99.7±0.6 | 47.6±4.5 |
| A1 | 75.1±5.1 | 12.8±1.8 | 99.2±0.8 | 98.1±1.6 | 99.3±0.5 | 97.4±0.4 |
| BF1 | 79.5±0.8 | 99.6±0.1 | 36.2±4.3 | 99.8±0.6 | 98.7±5.1 | -25.5±1.1 |
| BF3 | 80.2±4.9 | 99.0±0.4 | 99.7±0.8 | 99.4±8.4 | 99.4±0.5 | 95.3±7.9 |
| BF4 | 85.7±5.0 | 99.3±0.2 | 83.7±3.4 | 97.3±5.5 | 95.1±5.0 | -60.4±3.3 |
| BF5 | 95.5±3.6 | 12.9±1.8 | 00.0±11.4 | 99.9±5.8 | 99.4±0.2 | -95.2±1.2 |
| BF6 | 99.5±1.0 | 84.9±2.0 | 87.6±11.1 | 99.6±1.6 | 99.4±0.4 | 91.2±3.1 |
| BF9 | 78.3±1.8 | 99.0±1.9 | 95.8±1.7 | 99.9±8.8 | 97.3±0.9 | 89.9±6.3 |
| ZF1 | 43.0±6.0 | 96.8±3.1 | 95.7±2.6 | 97.1±5.2 | 73.7±4.7 | 92.9±4.4 |
| ZF2 | 63.4±2.9 | 72.1±4.0 | 127.4±12.9 | 99.7±0.2 | 78.4±0.6 | -113.1±11.6 |
| Growth control | -62.6±6.8 | | | -19.6±6.7 | | |
| Negative control | 0 | | | 0 | | |
| Positive control | 99.8±0.2 | | | 99.3±0.5 | | |
